# Apigeninidin chloride disrupts *Toxoplasma gondii* Mitochondrial membrane potential and induce reactive oxygen species and metabolites production

**DOI:** 10.3389/fcimb.2024.1368019

**Published:** 2024-11-11

**Authors:** Miya Janelle Moon, Japhet Senyo Kamasah, Homa Nath Sharma, Boakai K. Robertson, Daniel A. Abugri

**Affiliations:** ^1^ Department of Biological Sciences, College of Science, Technology, Engineering and Mathematics, Alabama State University, Montgomery, AL, United States; ^2^ Microbiology Ph.D. Program, College of Science, Technology, Engineering and Mathematics, Alabama State University, Montgomery, AL, United States; ^3^ Laboratory of Ethnomedicine, Parasitology and Drug Discovery, College of Science, Technology, Engineering and Mathematics, Alabama State University, Montgomery, AL, United States

**Keywords:** 3-DAs, *T. gondii*, tachyzoites, *in vitro*, mitochondrial membrane potential, reactive oxygen species, oxidative-stress metabolites

## Abstract

**Introduction:**

Apigeninidin chloride (APi) is a form of 3-deoxyanthrocyanidins (3-DAs) abundantly produced by the red *Sorghum bicolor* plant. It has been previously reported to be effective against *Toxoplasma gondii* (*T. gondii*) tachyzoites grown *in vitro* with less cytotoxic effect. However, its possible mechanism(s) of action has not been elucidated. Biochemically, we discovered that APi induced high reactive oxygen species (ROS) and mitochondria superoxide (MitoSOX) productions in tachyzoites, leading to mitochondrial membrane potential (MMP) disruption *in vitro*.

**Methods:**

To confirm our biochemical results at the molecular level, we performed a liquid chromatography-mass spectrometry (LC-MS) analysis on APi-treated parasites to assess any metabolite and lipid alterations often associated with high ROS/MitoSOX production in cells.

**Results:**

Noteworthy is that we detected several important oxidative stress-induced metabolites such as hexanal, aldehydes, methyl undeo10-enoate, butadiynyl phenyl ketone, 16-hydroxyhexadecanoic acid (16-OH, 16:0), 2-hydroxytricosanoic acid (C23:0; O), 3-oxodecanosanoic acid (C22:1; O), 2-hydroxypropylsterate, and furan fatty acids F6 (19FU-FA).

**Discussion:**

These metabolites are associated with lipid, protein, and nucleic acid disruptions. Using atovaquone (Atov) as a control, we observed that it disrupted intracellular tachyzoites’ mitochondrial membrane potential, increased ROS and MitoSOX production, and altered metabolite and lipid production similar to what was observed with our experimental compound APi. Overall, our results indicated that APi targets *T. gondii* tachyzoite growth through inducing oxidative stress, mitochondrial dysfunction, and eventually parasite death.

## Introduction

*T. gondii* is a common zoonotic protozoan parasite with wider host infections. The parasite’s seroprevalence has been reported in North America, Asia, Africa, Europe, and the Middle East in pregnant women, domestic fields, and food-bound animals (Dubey, 1996; Hussien et al., 2017; Cong et al., 2018; Bigna et al., 2020; Gong et al., 2020; Montazeri et al., 2020; Silva-Díaz et al., 2020; Dubey et al., 2021; Andreopoulou et al., 2023; Asgari et al., 2023; Cañedo-Solares et al., 2023; Hosseini et al., 2023; Kazemi et al., 2023; Mazuz et al., 2023; Otu-Bassey et al., 2023; Paştiu et al., 2023; Rizwan et al., 2023). Most of the underlying causes of human infection of *T. gondii* are through the consumption of contaminated tissue cyst meat, food, and fruits (Dubey, 1996; CDC, 2023). The most susceptible patients to *T. gondii*’s devastating effects are those with acquired immunodeficiency syndrome (AIDS) (Lee and Lee, 2017), pregnant women, cancer patients, and organ and tissue recipients (Bigna et al., 2020; Asgari et al., 2023; Kazemi et al., 2023; Otu-Bassey et al., 2023).

Currently, the standard treatment for toxoplasmosis is pyrimethamine plus sulfadiazine (PS), which targets *T. gondii*’s folate biosynthesis pathway (Shiojiri et al., 2019; Shammaa et al., 2021). In the event of resistance emergence to PS combination therapy, trimethoprim-sulfamethoxazole (TMP-SMZ), pyrimethamine and clindamycin, azithromycin plus clindamycin, atovaquone, and spiramycin are used as alternative drugs for treatment (Shiojiri et al., 2019; De Lima Bessa et al., 2021; Shammaa et al., 2021; CDC, 2023). Although these drugs are known to be effective against the tachyzoite stage, there are several drawbacks for use globally. Specifically, the challenges are (1) high toxicity at repeated high doses, (2) expensiveness, and (3) being not globally approved as a standard choice of treatment against *T. gondii* illness in patients (Julliac et al., 2010; McCarthy, 2015; Neville et al., 2015; Alday and Doggett, 2017; Ben-Harari et al., 2017; Shiojiri et al., 2019).

Oxidative stress is a very important biological process used to decipher the various harmful processes resulting from an imbalance between excessive formation of reactive oxygen species, superoxide, drugs, and limited antioxidant defenses in host cells, organs, and tissues (Turrens, 2003). It has been well established that intracellular redox signaling can occur as a result of changes in the steady-state concentration of the oxidants present (Dröge, 2002). Furthermore, any unsteady increase in the steady-state amounts of these oxidants can cause free radical-mediated chain reaction formation in the cell. These radicals formed can directly or indirectly target proteins (Stadtman and Levine, 2000), lipids (Rubbo et al., 1994), polysaccharides (Halliwell, 1995), and nucleic acids (e.g., DNA and RNA) (Richter et al., 1988; LeDoux et al., 1992; Turrens, 2003).

Drugs such as artemisinin, dihydroartemisinin, chloroquine, atovaquone, quinine, and artemether (Srivastava et al., 1997; Wenger et al., 2013; Egwu et al., 2021) and other new inhibitors of *T. gondii* such as hydroquinone (Huffman et al., 2022), curcumin (Das et al., 2008), and auranofin have been discovered to inhibit parasite proliferation either *in vitro* or *in vivo* (Wenger et al., 2013; Andrade et al., 2014). These compounds’ mechanisms of actions have been identified to perturb the redox signaling of the parasite by creating redox homeostasis imbalance in the parasite and eventually leading to parasite death (Wenger et al., 2013).

In our previous studies, 3-deoxyanthocyanindins have been screened against intracellular *T. gondii* growth *in vitro* (Abugri et al., 2016, 2017). Interestingly, the compounds had high selectivity indices with minimal cytotoxic effects on human foreskin fibroblast (HFF) *in vitro* (Abugri et al., 2016, 2017). Although these compounds have been found to be effective and safe *in vitro*, their possible mechanism(s) of action is still yet to be elucidated. Little has been known about their effect on parasites’ reactive oxygen species (ROS), mitochondrial superoxide (MitoSOX) production, and mitochondrial membrane potential (MMP) disruption. This research was conducted to assess APi’s effect on ROS and MitoSOX production, mitochondrial membrane potential integrity, and molecular metabolite production in *T. gondii* tachyzoites.

## Materials and methods

Apigeninidin chloride (APi) (10 mg) was obtained from Extra Synthase, Texas, U.S.A, where its main headquarter is in Cedex, France. APi was dissolved using dimethyl sulfoxide (DMSO). Azithromycin (CAS 83905–01-5) was obtained from Santa Cruz Biotechnology, located in Dallas, Texas. Azithromycin (AZ) of 25 mg (Lot D2020) was dissolved in DMSO (Lot No.: J2319), and atovaquone was obtained from Sigma Aldrich, USA, and stored in the refrigerator at −20°C. DMEM 1× (500 mL), Hi Bovine Serum (HBS), antibiotic [penicillin streptomycin–amphoteric B (PSA)], trypsin EDTA, and PBS were obtained from Thermo Fisher Scientific Inc., U.S.A. The percent of DMSO in the media was 0.1%.

### Host cell and parasite maintenance

hTERT cells (obtained from Distinguished Professor Silvia NJ Moreno from the University of Georgia, Athens, GA) were cultured in a T25 flask and supplemented with DMEM containing 5% FBS and 1% penicillin–streptomycin–amphoteric B (PSA). The RH-RFP *T. gondii* tachyzoites were added to confluent host cells and allowed to become confluent. Tachyzoites were purified from host cells using a 29-gauge needle with a 3-µm–5-µm filter for the mitochondrial superoxide, reactive oxygen species, and mitochondrial membrane potential integrity test.

### Extracellular reactive oxidative species production

To assess the ROS activity of APi, we extracted extracellular parasite (RH-wild type) type I strain (1 × 10^6^ tachyzoites), and 100 µL of this parasite count was added to black flat-bottom 96-well plates (Costar, Corning Inc., NY, USA). Subsequently, APi of concentrations 1.56 µM and 50 µM was added to the wells. The plates were incubated at 37°C with 5% CO_2_ for 30 min. At 30 min, 10 µL of 5 µM oxidative stress-orange reagent (CellROX™ Orange) dye was added to each well in the dark, covered with aluminum foil, and incubated at 37°C for 15 min according to the CellROX™ Orange reagent (C10443) protocol (Invitrogen, USA). The treated plates’ fluorescence intensities were taken using a Tecan 200F Infinite microplate reader with excitation/emission wavelengths set at 560 nm/635 nm, respectively (Sharma et al., 2023).

### APi induction of intracellular reactive oxidative species production

To assess whether APi and atovaquone (Atov) had any intracellular *T. gondii* tachyzoite ROS activity, we seeded approximately 1 × 10^3^ hTERT cells into black flat-bottom 96-well plates (Costar, Corning Inc., NY, USA) and allowed to grow to 95% confluency. Old media were removed and replaced with new growth media followed by addition of 1 × 10^6^ tachyzoites of RH-wild type, type I strain (200 µL/well) and incubated at 37°C with 5% CO_2_ for 2 h. The plates were washed thrice with 1× PBS, and 1.56-µM and 50-µM concentrations of APi and Atov were added to the intracellular parasites in designated wells. The plates were incubated at 37°C with 5% CO_2_ for 30 min. At 30 min, 50 µL of 5 µM oxidative stress-orange reagent (CellROX™ Orange) dye was added to each well and incubated in the dark (wrapped with aluminum foil) at 37°C for 30 min according to the CellROX™ Orange reagent (C10443) protocol (Invitrogen, USA; Sharma et al., 2023). Fluorescence intensities of wells were recorded at 560 nm/635 nm, respectively, with a Tecan 200F Infinite microplate reader (Abugri et al., 2023; Sharma et al., 2023).

### Extracellular mitochondrial superoxide production assay

Approximately 1 × 10^6^ tachyzoites/100 μL of *T. gondii* RH-W (wild-type) strain was added to each well of black flat-bottom 96-well plates. 50 µM of APi and 500 μM H_2_O_2_ as a positive control, and media only as a negative control were added to the wells. The plates were incubated at 37°C with 5% CO_2_ for 3 h. After 3 h, 50 μL of 5 μM of MitoSOX™ reagent was carefully added into each well, wrapped with aluminum foil to avoid light interferences, and incubated at 37°C for 30 min according to the protocol for the MitoSOX™ Red mitochondrial superoxide indicator (M36008) provided by Invitrogen, USA (Sharma et al., 2023). The fluorescence intensities of wells were measured using a Tecan 200F Infinite microplate reader with 485-nm excitation and 535-nm emission. Experiments were conducted six times (n = 6) using single wells (Sharma et al., 2023).

### APi and Atov induction of intracellular mitochondrial superoxide production

For intracellular mitochondrial superoxide assessment, 1 × 10^6^ tachyzoites/200 μL of *T. gondii* RH-W (wild type) strain were added to each well of black flat-bottom 96-well plates containing 1 × 10^3^ confluent hTERT cells. The plates were incubated at 37°C with 5% CO_2_ for 2 h and washed three times with 1× PBS. We then added 50 μM and 1.5 μM of APi and Atov, respectively, in designated wells. Media only were added to the designated wells as a negative control. The plates were incubated at 37°C with 5% CO_2_ for 3 h. After 3 h, 50 μL of 5 μM of MitoSOX™ reagent was carefully added into each well, wrapped with aluminum foil to avoid light interferences, and incubated at 37°C for 30 min according to the protocol for the MitoSOX™ Red mitochondrial superoxide indicator (M36008) provided by Invitrogen, USA (Sharma et al., 2023; Abugri et al., 2023). The fluorescence intensities of wells were measured using a Tecan 200F Infinite microplate reader with excitation and emission wavelengths set at 485 nm and at 535 nm, respectively. Experiments were performed in triplicates (n = 3).

### APi’s effect on extracellular *T. gondii* mitochondrial membrane potential

In this assay, *T. gondii* RH wild-type I strain tachyzoites (1 × 10^6^/50 μL) were seeded into black 96-well plates (Costar, Corning Inc., NY, U.S.A). 50 μL of 50 μM of APi, 50 μL of 50 μM of carbonyl cyanide *m*-chlorophenyl hydrazone (CCCP) as a positive control (obtained from Alfa Aesar, Haverhill, MA, USA), and assay buffer (Hanks’ balanced saline solution (HBSS) without phenol red) as the negative control were added to each designated well and incubated for 8 h at 37°C with 5% CO_2_ (Huffman et al., 2022). At 8 h, 10 μL of a cationic probe JC-1 (Thermo Fisher Scientific, Waltham, CA, USA) dye was added to the wells, and the plates were covered with aluminum foil to avoid photolysis and allowed to incubate for 45 min. At 45 min, the plates were centrifuged at 12°C and 2,000 rpm for 5 min, the supernatant was removed, and 100 μL of assay buffer was added to each well. The process was repeated once more and the pellet containing *T. gondii* tachyzoites was suspended with 100 μL of assay buffer, with the fluorescence read at 485 nm/535 nm and 560 nm/635 nm, respectively (Huffman et al., 2022; Sharma et al., 2023). Experiments were performed six times using individual wells (n = 6).

### APi and Atov effect on intracellular *T. gondii* mitochondrial membrane potential

1 × 10^3^ hTERT cells were grown in black flat-bottom 96-well plates (Costar, Corning Inc., NY, USA) and incubated at 37°C with 5% CO_2_ for 72 h to confluence. The plates were washed thrice with 1× PBS, and 200 µL of 1 × 10^6^ freshly purified RH-W (wild type) *T. gondii* type 1 strain tachyzoites was seeded into it and incubated again at 37°C with 5% CO_2_ for 2 h for parasite invasion. Extracellular parasites were washed off thrice with 1× PBS, and 50-µM and 1.5-µM concentrations of apigeninidin (APi) and atovaquone (Atov) were added to designated wells (100 µL/well). Carbonyl cyanide p-trifluoro-methoxyphenyl hydrazone (FCCP) and media only were used as positive and negative controls, respectively, and incubated for 8 h at 37°C with 5% CO_2_, as previously described (Abugri et al., 2023; Sharma et al., 2023). 100 µL of 1 µM JC-1 solution was added to each well and incubated for 20 min at 37°C with 5% CO_2_ in the dark and measured using a Tecan 200F Infinite microplate reader at excitation/emission wavelengths set at 485/530 for monomer and 535/590 for aggregates, respectively (Abugri et al., 2023; Huffman et al., 2022; Sharma et al., 2023). Micrographs of parasites’ mitochondria membrane impairment were taken using an EVOS fluorescent microscope.

### Oxidation-induced metabolite extraction

hTERT cells were grown on a T25 flask to become 90% confluent. *T. gondii* type 1 strain (RH-RFP tachyzoites with a final concentration of 4.22 × 10^4^ parasites per mL) was added to the T25 flask afterward, and then 50 µL of APi, AZ, and Atov was added to compound-designated flasks. Next, the T25 flasks were incubated for 24 h and 48 h at 37°C with 5% CO_2_. At each time point, the flasks were removed from the culture incubator and chilled under ice for 5 min. Host cells containing parasites were scraped, and then the parasites were passed through a 25/29-gauge needle followed by 3-micron filter. Parasite suspension was centrifuged at 12,000 rpm, at 12°C for 5 min. Supernatants were carefully removed from the cell pellets. The process was repeated twice, and the pellets dissolved in ice-chilled methanol–chloroform and vortexed for 5 min, followed by centrifugation. The extracts were stored in a −20°C refrigerator overnight prior to LC-MS/MS analysis at the mass spectrometry facility at Auburn University.

### LC-MS/MS analysis

Analysis was performed on a Vanquish UHPLC system (Thermo Fisher, USA) coupled with a quadrupole orbitrap mass spectrometer (Orbitrap Exploris 120, Thermo) with electrospray ionization (H-ESI) in positive and negative modes using Xcalibur software (V4.4.16.14). Approximately 10 μL of the *T. gondii* tachyzoite metabolite extract was injected onto a C18 column (ACQUITY UPLC^®^ BEH C18, 1.7 µm, 2.1 × 50 mm, Waters) with a 200-μL/min flow rate of a mobile phase of solution A (0.1% formic acid in 50% water and 50% methanol) and solution B (50% acetonitrile and 50% isopropanol with 0.05% formic acid) beginning at 30%B to 50%B in 1 min followed by a linear gradient to 100%B in 13 min, held for 3 min, and then returning to 30%B and 3 min of re-equilibration (total time of 21 n. The spray voltage was set at 3.5 kV in positive mode and 3.0 kV in negative mode. The samples were injected twice, one for each mode. The sheath gas was set at 30, aux gas at 20, and sweep gas at 0 (all arbitrary units) with vaporizer temperature and ion transfer tube temperature of 300°C and 350°C, respectively. The orbitrap resolution was set to 120,000 for MS and 15,000 for DDA MS/MS with four dependent scans with an intensity threshold of 20,000, auto dynamic exclusion, and a targeted exclusion mass list based on blank injections. EASY-IC was on for the MS scan with range 115 Da–1,000 Da. Collision energy was normalized and stepped at 10, 40, and 100 with the max injection time set to auto (Sharma et al., 2023).

### Data processing

Samples were processed with Compound Discoverer 3.2 using the natural products and untargeted Metabolomics workflow with the Carotenoids Database, Human Metabolome Database, and Lipid MAPS.

### Statistical analysis

The graphs/heat maps were obtained using GraphPad Prism software version 9.4.1. A one-way ANOVA was used to determine any statistical difference by using the Holm–Sidak multiple-comparisons test on treated samples relative to the controls or in other cases different doses/treatments (Huffman et al., 2022). Statistically significant difference was determined with the alpha value set to 0.05.

## Results

### Reactive oxidative species production

**Figure 1:**
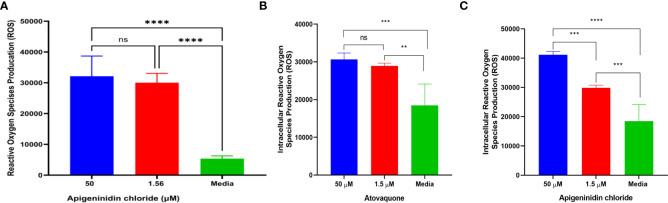
**(A)** Apigeninidin chloride effect on extracellular *T. gondii* tachyzoite reactive oxygen species. **** represents means plus standard deviation of six independent experiments performed in single wells with *p* < 0.0001, respectively. ns, no significant differences. **(B, C)** represent intracellular ROS production induced by atovaquone and apigeninidin chloride, respectively. For **(B)**, ** and *** represent p values = 0.012 and 0.0003, respectively. For **(C)**, *** and **** represent *p* values = 0.0005 and <0.0001, respectively. Experiments are performed in triplicates (n = 3).

In this study, we report the effect of APi on extracellular *T. gondii* tachyzoite reactive oxidative species (ROS) production in a dose–dependent manner (**Figure 1A**). There was a significant difference in ROS production between the 50 µM of APi-treated parasites and the [control (media only)] with *p* < 0.0001. Similarly, the parasites treated with a lower concentration of APi (1.56 µM) were also statistically significant compared with the control group with *p* value less than 0.0001. Contrarily, the higher concentration of APi and the lower concentration of APi were not statistically different (*p* > 0.05).

Intracellular analysis of the Atov effect on *T. gondii* tachyzoite ROS production showed that the concentrations of 50 µM and 1.5 µM tested for Atov were statistically significantly different from the media with *p* values = 0.012 and 0.0003 (**Figure 1B**). However, we did not detect any statistically significant difference between the 50-µM and 1.5-µM concentrations tested for Atov. This observation could be due to host cell interferences. However, further study is needed to understand this trend. With APi, we found a significant difference between the 50-µM and 1.5-µM concentrations and between the 1.5-µM and media concentrations, respectively (*p* = 0.0005). Also, there was a significant difference between 50 µM and media at (*p* < 0.0001) (**Figure 1C**).

### Mitochondria superoxide production

**Figure 2:**
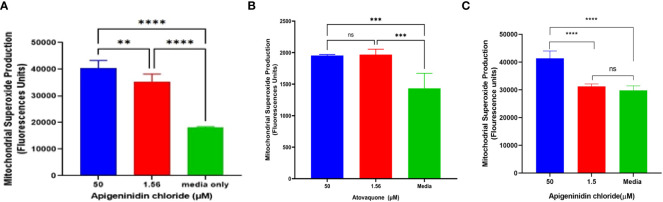
**(A)** Apigeninidin chloride effect on *T. gondii* mitochondrial superoxide production. APi (50 μM and 1.56 μM) and media-only effects on mitochondrial superoxide production (MitoSOX) in *T. gondii* tachyzoites. Data are presented as means plus standard deviation of six independent experiments performed in single wells with *p* < 0.001 (**) and *p* < 0.0001 (****). **(B, C)** represent intracellular MitoSOX production in *T. gondii* tachyzoites induced by atovaquone and apigeninidin chloride, respectively. On **(B)**, *** represents *p* value = 0.012, whereas **** represents *p* values < 0.0001 on **(C)** and ns, no significant differences on both figures. Experiments are performed in triplicates (n =3).

APi was evaluated against *T. gondii* tachyzoite mitochondrial superoxide production in a dose-dependent manner (**Figure 2A**). Noteworthy is that we observed a significant difference between the 50-µM and 1.56-µM concentrations of APi-treated parasites (*p* < 0.01), 50 µM versus media (*p* < 0.0001), and 1.56 µM versus control (*p* < 0.001) (**Figure 2A**).

In the intracellular MitoSOX analysis of the effect of Atov on *T. gondii* tachyzoites, we observed a significant difference between the 50 µM and 1.5 µM and the media (acting as a negative control) (**Figure 2B**). There was no difference between the 50-µM and 1.5-µM treatment of atovaquone. This might be attributed to host cell interference. However, further study is needed to decipher this trend. APi treatment showed statistical difference between the 50 µM and 1.5 µM and between 50 µM and media (**Figure 2C**) with *p* < 0.0001). No significance difference was found between 1.5 µM and the control (**Figure 2C**).

### Mitochondria membrane potential dysfunction

**Figure 3:**
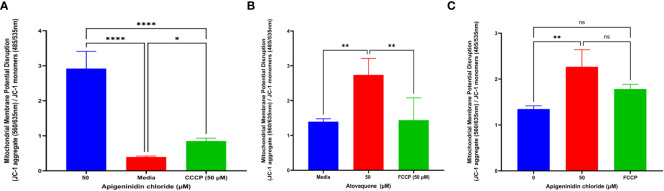
**(A)** Apigeninidin chloride effect on *T. gondii* mitochondria membrane potential disruption. Quantitative pattern of *T. gondii* mitochondria membrane potential disruption. * and **** represent means plus standard deviation of six independent experiments performed in single wells with *p* < 0.05 and *p <*0.0001, respectively. **(B, C)** Atovaquone and apigeninidin chloride effect on the intracellular *T. gondii* mitochondria membrane potential. ** represents means plus standard deviation of three independent experiments performed in single wells with *p* < 0.05; ns, no significant differences. Experiments are performed in triplicates (n = 3).

It was discovered that APi affected extracellular *T. gondii* tachyzoites’ mitochondrial membrane potential in a dose-dependent manner (**Figure 3A**). Statistically, there was a significant difference between the 50 µM of APi and the negative control treatments (*p* < 0.0001). Furthermore, we also observed a statistical difference between 50 µM and the positive control [CCCP (50 µM) with (*p* < 0.0001)]. CCCP was also statistically different from the control with *p* < 0.05.

Atov and APi had an effect on intracellular *T. gondii* mitochondrial membrane potential, as shown in **Figures 3B** and **C**. We discovered a significant difference between media and 50 µM of Atov tested (*p* < 0.05). Similarly, there was a statistical difference between 50 µM of Atov and the FCCP used as a standard compound with the same p value. For the APi treatment, we detected a difference between the media and the 50-µM concentration (*p* < 0.05). However, no significant difference was found between the 50-µM treatment of APi and the 50-µM FCCP (**Figure 3C**). This could imply that both compounds tested at the same concentration exerted the same intracellular strength on mitochondria membrane potential disruption.

To validate our high content results reported in the graphs (**Figures 3B, C**), we captured the red fluorescence and green fluorescence micrographs which indicate healthy and compromised mitochondria membranes, respectively, as shown in **Supplementary Figures 1A, B**. The micrographs indicated that there was an MMP disruption in intracellular *T. gondii* parasites. Furthermore, the higher concentration of 50 µM used for FCCP, Atov, and APi was observed to show brighter-green fluorescence than the 1.5-µM concentration used for APi and Atov (**Supplementary Figures 1A, B**).

### Oxidative stress-induced lipid peroxidation expression

**Figure 4:**
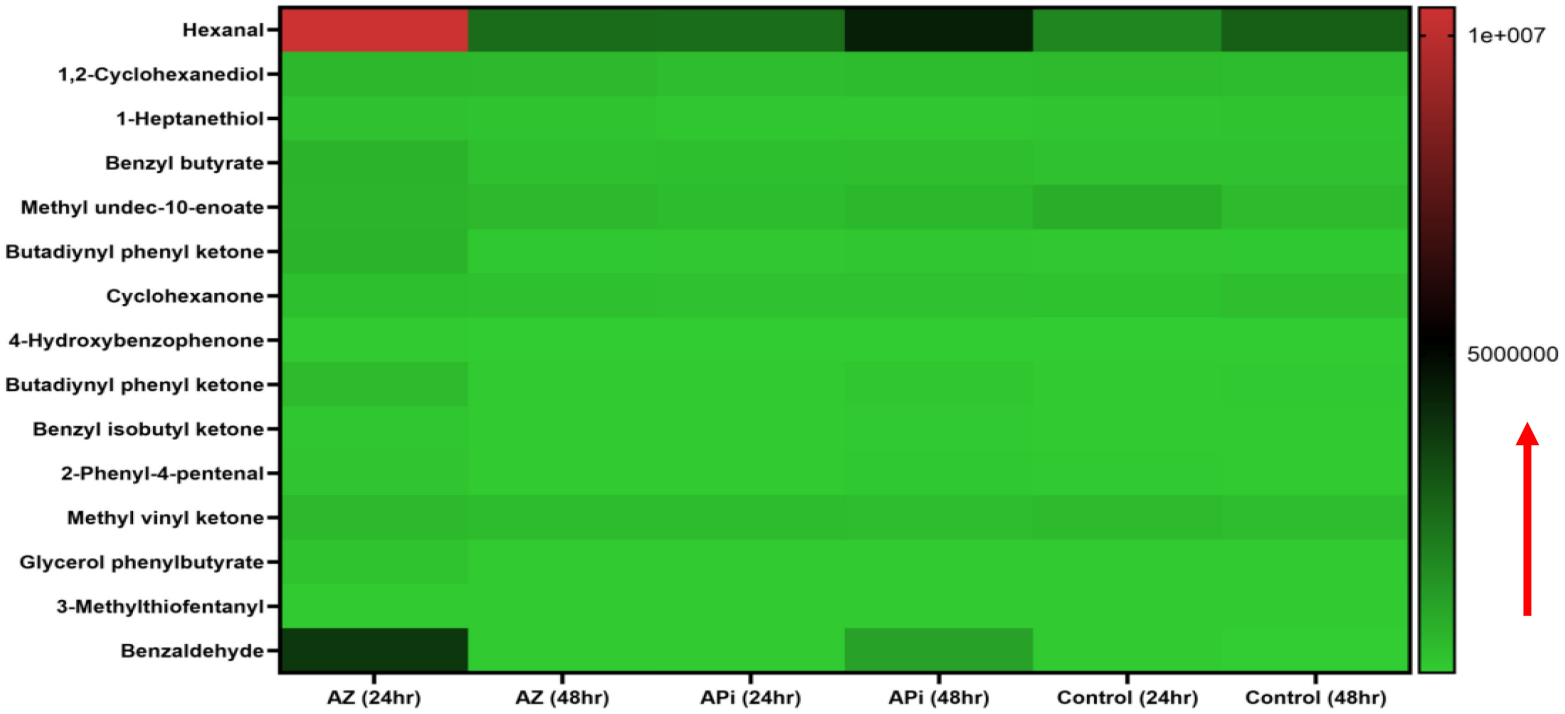
Heat map of ROS/MitoSOX-induced non-lipid metabolites expressed in *T. gondii* tachyzoites. Parasites were treated with 50 μM of APi and AZ for 24 h and 48 h, respectively. Data are presented as a means of duplicate independent experiments. The red arrow indicates metabolite production from a scale of 0 being underproduced, 5.0 × 10^6^ being moderately produced, and 1 × 10^7^ being highly produced in parasites.

In the metabolomics analysis, 16 metabolites were identified as presented in (**Figure 4**). The most highly lipid-peroxidant metabolites formed as a result of the elevated ROS and MitoSOX production were hexanal and benzaldehydes. These two metabolites produced by the APi-treated parasites were observed to increase in a time-dependent manner. More specifically, at 48 h, hexanal and benzaldehyde production was greater than 5 × 10^6^ and 4 × 10^6^ in a scale of 0 to 1 × 10^7^ range (**Figure 4**). The trend of hexanal production was similar in the APi-treated and control groups at both 24 h and 48 h.

AZ treatment at 24 h was observed to induce high production of methyl undeo-10-enoate, butadiynyl phenyl ketone, hexanal, and benzaldehydes compared with the 48-h treatment of AZ and the negative control group (media only) in intracellular parasites *in vitro* (**Figure 4**). Hexanal and benzaldehyde production in parasites treated with AZ were observed to range in a scale of approximately 1 × 10^7^ and 5 × 10^6^, respectively, as indicated by the scale depicted in **Figure 4**. Strangely, we observed hexanal production to increase at 48 h in the control group.

**Figure 5:**
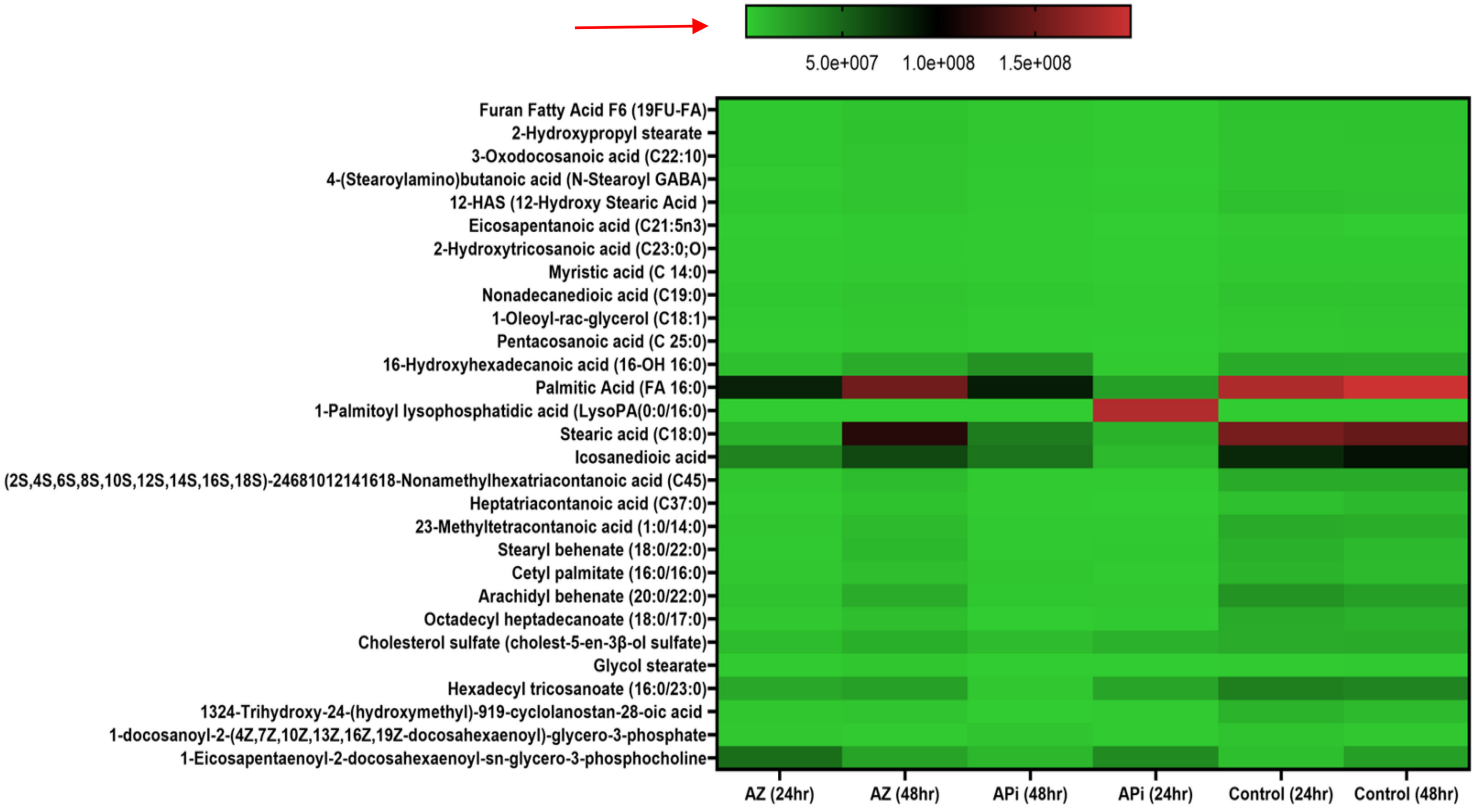
Heat map of ROS/MitoSOX-induced lipid metabolite production in *T. gondii* tachyzoites. Parasites were treated with 50 μM of APi and AZ versus controls (media-treated parasites) for 24 h and 48 h, respectively. Data are presented as means of duplicate independent experiments. The red arrow indicates metabolite production from a scale of 5.0 × 10^7^ being underproduced, 1.0 × 10^8^ being moderately produced, and 1.5 × 10^8^ being highly produced in parasites.

Significantly, in our lipidomic peroxidation LC-MS analysis, we observed an increase in certain metabolite production in both APi- and AZ-treated groups in a time-dependent manner (**Figure 5**). Some of the ROS/MitoSOX-induced lipid metabolites detected were 16-hydroxyhexadecanoic acid (16-OH, 16:0), 2-hydroxytricosanoic acid (C23:0; O), 3-oxodecanosanoic acid (C22:1; O), 2-hydroxypropyl stearate, furan fatty acids F6 (19 FUFA), stearic acid, icosanedioic acid, and palmitic acid. The relative areas of abundance of the metabolites presented in **Figure 4** are presented in the **Supplementary Data** section as **Figure 4 Supplementary Data**.

Furthermore, in AZ- and APi-treated parasites, lipid 1-eicosapentaenoyl-2-docosahexaenoyl-sn-glycero-3-phosphocholine production was observed to decrease at 48 h with a scale less than 5.0 × 10^7^ (**Figure 5**). However, in the control (media-only treatment) group, we observed an increase in 1-eicosapentaenoyl-2-docosahexaenoyl-sn-glycero-3-phosphocholine production at 48 h (**Figure 5**). Also, uniquely, APi treatment caused a decrease of cholesterol sulfate production at 48 h than 24 h of treatment of the APi and control groups. There was a decrease in hexadecyl tricosanoate production in APi-treated parasites and the control group from 24 h to 48 h. Another important finding was that 1-palmitoyl-lysophosphatidic acid was highly decreased at 48 h of treatment with APi compared with 24 h with APi, as shown on the color scale. There was no change of its production in the AZ and control groups (**Figure 5**). One unique observation about the AZ-treated group was the high production of nonamethylhexatriacontanoic acid, 16-hydroxyhexadecanoic acid, stearic acid, cholesterol sulfate, heptatriacontanoic acid, 23-methyltetracontanoic acid, arachidyl behenate, and stearyl behenate at 48 h but not with the APi and negative control groups with a scale greater than 5.0 × 10^7^ and closer to 1.5 × 10^8^ (**Figure 5**). The detected metabolites with their molecular weights and relative areas of abundance presented in **Figure 5** are presented in the **Supplementary Data** section as **Figure 5 Supplementary Data**.

**Figure 6:**
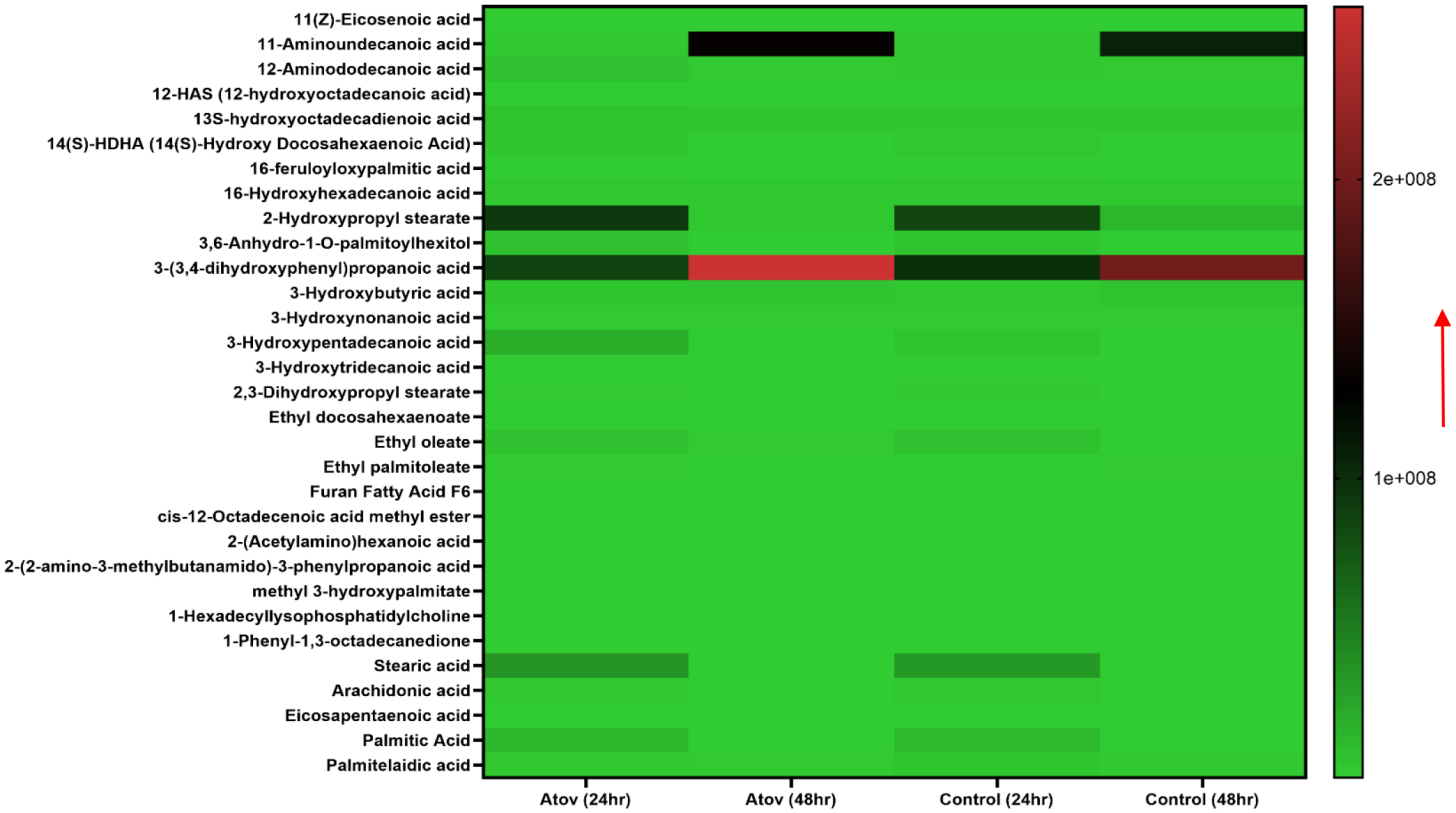
Heat map showing Atov-induced ROS/MitoSOX lipid and lipid-oxidized metabolite production in *T. gondii* tachyzoites. Parasites were treated with 50 μM of Atov versus controls (media-treated parasites) for 24 h and 48 h, respectively. Our data are reported as means of duplicate independent experiments. The red arrow indicates metabolite production from a scale of 1.0 × 10^8^ being underproduced and 2 × 10^8^ being highly produced in the parasites.

Testing Atov as a standard drug, which is known to target the mitochondria, showed the following fatty acids and oxidized lipids; 11-aminoundecanoic acid, 12-aminododecanoic acid, 13S-hydroxyoctadecadienoic acid, 14(S)-HDHA (14(S)-hydroxy docosahexaenoic acid), 16-feruloyloxypalmitic acid, 2-hydroxypropyl stearate, 3, 6-anhydro-1-O-palmitoylhexitol, 3-(3,4-dihydroxyphenyl)propanoic acid, 3-hydroxybutyric acid, 3-hydroxynonanoic acid, 3-hydroxypentadecanoic acid, 3-hydroxytridecanoic acid, 2,3-dihydroxypropyl stearate, ethyl docosahexaenoate, ethyl oleate, ethyl palmitoleate, cis-12-octadecenoic acid methyl ester, 2-(acetylamino)hexanoic acid, 2-(2-amino-3-methylbutanamido)-3-phenylpropanoic acid, methyl 3-hydroxypalmitate, 1-hexadecyllysophosphatidylcholine, and 1-phenyl-1,3-octadecanedione (**Figure 6**).

**Figure 7:**
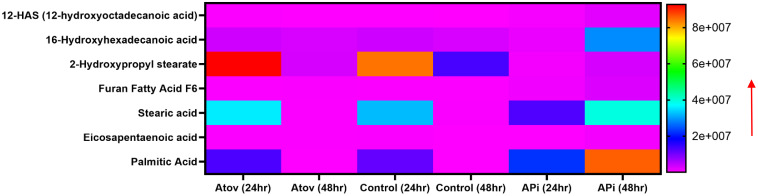
Heat map comparison of AT- and API-induced ROS/MitoSOX common lipid metabolite production in *T. gondii* tachyzoites. Parasites were treated with 50 μM of APi and Atov versus controls (media-treated parasites) for 24 h and 48 h, respectively. Data are presented as means of duplicate independent experiments. The red arrow indicates metabolite production from a scale of <2.0 × 10^8^ being underproduced, between 4 × 10^7^ and 6.0 × 10^7^ being moderately produced, and 8 × 10^7^ being highly produced in parasites.

Palmitic acid, stearic acid, 2-hydroxypropyl stearate, and 16-hydroxyhexadecanoic acid were observed to decrease in production at 48 h of interaction with Atov compared with the 24-h interaction. The control at 24 h was slightly produced for palmitic acid, stearic acid, and 16-hydroxyhexadecanoic acid. Comparing Atov with APi, we observed that APi both had 12-HAS (12-hydroxyoctadecanoic acid), 16-hydroxyhexadecanoic acid, 2-hydroxypropyl stearate, furan fatty acid F6, stearic acid, eicosapentaenoic acid, and palmitic acid (**Figure 7**). These oxidized and non-oxidized lipids were observed to increase in production at 48 h in APi-treated parasites than those Atov treated (**Figure 7**).

Our mass spectrometry analyses of Atov-treated parasites showed some oxidized lipids and non-oxidized lipids that were similar to what was observed in APi-treated parasites (**Figure 7**).

**Figure 8:**
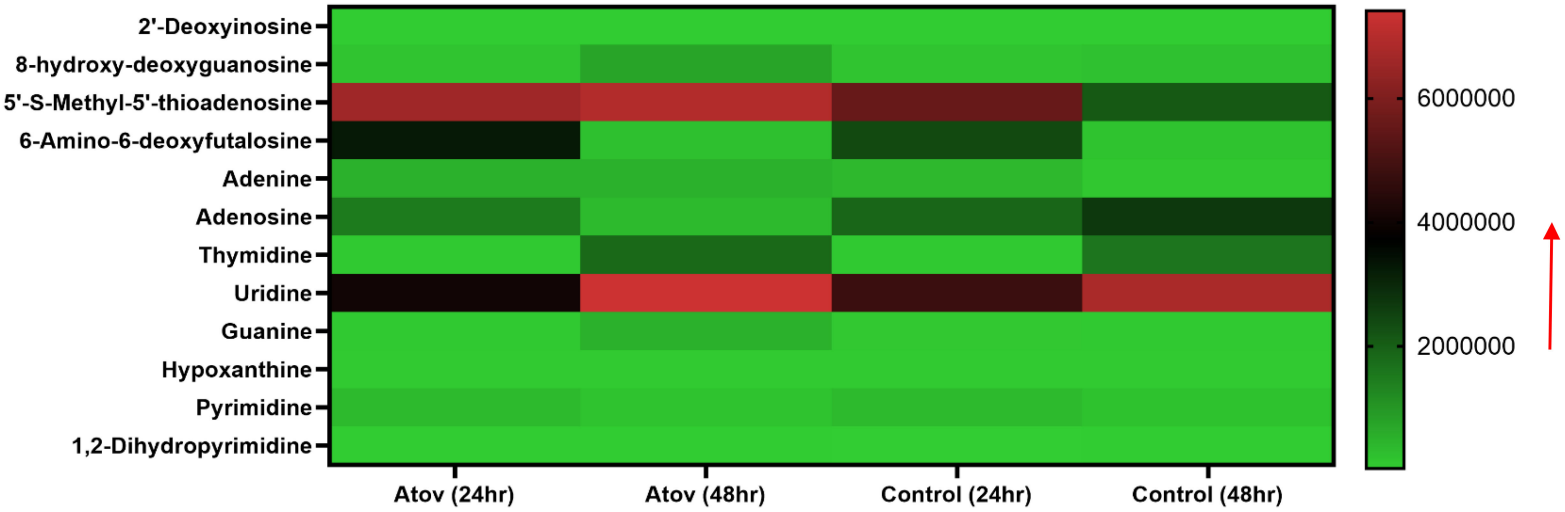
Heat map showing Atov-induced nucleic acid precursor alteration due to high ROS/MitoSOX production in *T. gondii* tachyzoites. Parasites were treated with 50 μM of Atov versus controls (media-treated parasites) for 24 h and 48 h, respectively. Data are given as means of duplicate independent experiments. The red arrow indicates metabolite production from a scale of 2.0 × 10^6^ being underproduced, 4.0 × 10^6^ being moderately produced, and 6 × 10^6^ being highly produced in the parasites.

Furthermore, we detected nucleotides and nucleosides such as 2′-deoxyinosine, 8-hydroxy-deoxyguanosine, 5′-S-methyl-5′-thioadenosine, 6-amino-6-deoxyfutalosine, adenine, adenosine, thymidine, uridine, guanine, hypoxanthine, pyrimidine, and 1,2-dihydropyrimidine (**Figure 8**).

Notable, Atov treatment caused 8-hydroxy-deoxyguanosine, thymidine, guanine, and 5-S-methyl-5′-thioadenosine production to increase at 48 h of interaction than 24 h of interaction (**Figure 8**).

## Discussion

Ocular toxoplasmosis and congenital toxoplasmosis continue to increase globally (Pinto-Ferreira et al., 2019; Arruda et al., 2021; Kamus et al., 2023). The most recommend drugs used for treatment of toxoplasmosis are pyrimethamine–sulfadiazine combination (Ben-Harari et al., 2017; Lee and Lee, 2017; Shiojiri et al., 2019; Shammaa et al., 2021; CDC, 2023). However, this combination has been reported to be ineffective in some patients. To overcome these drawbacks, drugs such as atovaquone, spiramycin, clindamycin, and azithromycin are used as alternative medication for the treatment of *T. gondii* infection (Shiojiri et al., 2019; Shammaa et al., 2021). Even with this alternative medication, there are still problems with toxicity issues generally reported globally and thus threaten the use of these medication singly or in combination. To avoid these toxicity issues that might have not been well investigated during the early discovery of these drugs, biochemical and metabolomics approaches have been developed and used to identify certain drug targets. Specifically, drugs such as atovaquone, chloroquine, and artemisinin and other new inhibitors (e.g., curcumin, artemether, and dihydroquinine) have been biochemically reported to cause mitochondrial membrane potential depolarization (MMP) and mitochondria dysfunction leading to high ROS and MitoSOX production in parasites and eventually causing parasites death (Egwu et al., 2011; Srivastava et al., 1997; Das et al., 2008; Wenger et al., 2013; Huffman et al., 2022).

In this study, we demonstrated for the first time that APi’s possible mechanism of action against intracellular *T. gondii* tachyzoite growth previously reported in our group (Abugri et al., 2016, 2017) was through excessive ROS and MitoSOX production resulting in mitochondrial membrane potential disruptions and eventually parasite death. The possible reasons for APi ROS, MitoSOX production, and MMP disruption might be attributed to its structural components such as (i) the presence of the hydroxyl groups and phenolic rings, making it highly antioxidant, and (ii) the presence of the chloride ion capable of attacking the cationic phenol group, which makes it more antioxidant and oxidant. Specifically, we reason that halide’s (Cl) presence in APi is acting as the oxidizing agent, which has the ability to cause oxidative stress on the parasite and eventually death. This has been reported as the mechanism of action of halides killing of bacteria (Wilson et al., 2020). Additionally, we believe that the presence of the phenols in APi makes it penetrate the membrane easily, intercalate and disrupt cellular membranes (e.g., mitochondrial membrane), and interact with proteins leading to essential proteins denaturation. It has been shown that cleaning reagents containing phenols exhibit these mechanisms of action (Wilson et al., 2020). However, further studies are required to confirm these assumptions.

Previous studies have reported that Atov’s structure containing the naphthoquinone group and chlorophenyl groups is the cause of its mechanistic properties such as mitochondrial membrane potential disruption and increase in ROS and MitoSOX production in parasites and cancer cells (Das et al., 2018; Ke et al., 2018; Alharbi et al., 2020; Coates et al., 2020; Egwu et al., 2021; Kapur et al., 2022; Fiorillo et al., 2016; Srivastava et al., 1997; Birth et al., 2014). Structurally, both APi and Atov have chloride and hydroxyl groups as part of their phenol-like and quinolone-like structures. Significantly, we observed a statistical difference between the 50 µM and the negative control (0 µM) effects on mitochondrial membrane potential disruption. Additionally, the higher concentration of 50 µM used for FCCP, Atov, and APi was observed to be brighter in the green fluorescence than the 1.5-µM concentration used for both APi and Atov (**Supplementary Figures 1A, B**). Also, of interest was the fact that lower concentrations such as 1.5 µM produced healthy mitochondrial membranes than morphology visibility, such as the higher concentration of 50 µM used in APi. This implies that the degree of MMP disruptions by APi is concentration dependent. Similar evidence was obtained for Atov tested as a standard positive control.

Both APi and Atov induced high ROS and MitoSOX production in intracellular *T. gondii* tachyzoites. It has been documented that Atov causes ROS/MitoSOX production in *P. falciparum* as well as cancer and human foreskin fibroblast cells (Das et al., 2018; Ke et al., 2018; Alharbi et al., 2020; Coates et al., 2020; Egwu et al., 2021; Kapur et al., 2022; Fiorillo et al., 2016). Thus, our compound (APi) exhibition of these two forms of reactive oxidative stress biomarkers on both extracellular and intracellular *T. gondii* implies that its mechanism of action may be similar to Atov that have some functional groups (e.g., chlorine and hydroxyl) and phenolic rings similar to APi. These findings confirmed previous studies of Atov having been known to affect mitochondrial complex III in *P. falciparum*, leading to electron transport chain disruption and ATP depletion (Srivastava et al., 1997; Egwu et al., 2021). However, little is known about APi exerting a similar mechanism of action. Thus, future work will be necessary to decipher whether APi has any direct effect on the mitochondrial complexes and electron transport chain disruption. Also, one thing not clear is that we tested only few oxidative stress biomarkers and observed these findings. We do not know whether there might be other modes of action contributing to these observations made. Thus, future studies will be required to assess other ROS species using subnanomolar concentrations of APi and Atov to see whether it is effective. This will be crucial to developing non-toxic and effective drug against toxoplasmosis.

Another interesting finding was that our study supports several findings that excessive ROS and MitoSOX production causes high free radical generations that target polyunsaturated fatty acids, proteins, and nucleic acids in cells (Riahi et al., 2010; Das and Roychoudhury, 2014; Yoshida et al., 2015; Waszczak et al., 2018). More specifically, the polyunsaturated fatty acids (PUFAs) disrupted by this redox oxidative stress are arachidonic and linoleic acids which can be oxidized into a racemic mixture of 13-hydroxy-9 Z, 11E-octadecadieonic acid, 13-hydroxy-8E,11E-octadecadienoic and 9-hydroxy-10E for arachidonic acid, and 12-E-octadecadienoic acid and 11-hydroxy-9Z,12-Z-octadecadienoic acid for linoleic acid (Riahi et al., 2010; Das and Roychoudhury, 2014; Yoshida et al., 2015; Waszczak et al., 2018). Also, another toxic compound such as 4-hydroxynonenal has been reported to occur in excessive ROS/MitoSOX-generated cells (Riahi et al., 2010; Das and Roychoudhury, 2014; Yoshida et al., 2015; Waszczak et al., 2018). These compounds could further interact with each other to produce more toxic compounds such as alkenes, alkenals, aldehydes, epoxy alcohols, epoxy ketones, hydroperoxides, and ketone lipid radicals (Das and Roychoudhury, 2014), which are toxic to the parasites and their environment and could eventually lead to *T. gondii* death. Significantly, we discovered that the APi-treated parasites resulted in increase of hexanal, benzaldehyde, methyl undeo-10-enoate, and butadiynyl phenyl ketone production in a time-dependent manner (**Figure 4**). These metabolites’ presence in media might cause alteration of parasites’ cellular function (e.g., survival, proliferation, efficient uptake of nutrient and metabolism, proper lipid and protein synthesis and functionality). It has been reported that aldehydes cause changes to cellular properties of cells and result in covalent-protein adduct formation (Grimsrud et al., 2008). Furthermore, aldehydes are amphiphilic in nature, easily transverse the cell membrane, and cause essential protein modifications in the cytoplasm and nucleus of cells (Negre-Salvayre et al., 2008). Therefore, the presence of benzaldehydes in the treated parasites is likely to cause protein modification in parasites and alteration of cellular function including survival, replication, and eventually parasite death. Alkanals are known to form adducts with amino acid acyl side chains especially cysteine, histidine, and lysine leading to modification of thiol and amino groups and the tertiary structure of protein at the cellular level (Pizzimenti et al., 2013). Thus, our findings with the hexanals are likely to cause cysteine thiol disruption leading to the production of sulfinic and sulphonic acids, which are biomarkers of oxidative protein damage (Poole et al., 2020; Lennicke and Cochemé, 2021; Murphy et al., 2022).

In contrast, AZ did not cause an increase of hexanal and benzaldehyde production in parasites at 48 h of treatment. This implies that AZ treatment does not induce ROS/MitoSOX in parasites and could be exerting other mechanisms of action (such as protein synthesis) against *T. gondii* growth, which is generally known. Using Atov, as the recommended standard, we show that it induces ROS/MitoSOX production in intracellular *T. gondii* parasites.

High ROS/MitoSOX production and mitochondria disruption have been associated with nucleic acid distortion (Murphy et al., 2022; Andrés et al., 2023). Interestingly, we detected 8-hydroxy-deoxyguanosine, 5′-S-methyl-5′-thioadenosine, 6-amino-6-deoxyfutalosine, and 1,2-dihydropyrimidine in Atov-treated parasites. Production of these metabolites suggests that the high ROS reported in our biochemical assay for Atov affected the nucleic acid and production of its bases in the parasites. This finding confirmed high ROS alteration of the double DNA strand and oxidation of the nucleoside and nucleotide (Murphy et al., 2022; Andrés et al., 2023). Also, it has been reported that high ROS production can result in amino acid disruption and modification (Kapur et al., 2022; Andrés et al., 2022). Here, we observed that Atov treatment caused some amino acid hydroxylation, sulfonation, and acetylation. The most prominent reactive species-induced amino acids detected were 3-hydroxy-3-methylglutaric acid, 3-(sulfooxy)-L-tyrosine, N(6)-[(indol-3-yl) acetyl]-L-lysine, S-[(1Z)-N-hydroxy-9-(methylsulfanyl)nonanimidoyl]cysteine, and S-Allyl-L-cysteine. Our data supported what has been reported about Atov’s effect on metabolite production and alterations of amino acid metabolism at 24 h of treatment in cancer cells (Kapur et al., 2022). Furthermore, according to Kapur et al. (2022), cysteine and histidine were not produced, indicating that the high ROS might have caused the production of alkanals; methylated, ally, hydroxyl, acetyl, and sulfoxyl cysteine; and other amino acids. These alkanals may have caused the thiol, amino group, and tertiary structure modification in the cell. Hence, we believe that a similar mechanism might have occurred in the Atov-treated parasites. This deduction requires future studies to ascertain this conjecture.

Distinctively, the excess ROS/MitoSOX produced by APi on the parasites induced lipids/fatty acid peroxidant generation [e.g., 16-hydroxyhexadecanoic acid (16OH,16:0), 2-hydroxytricosanoic acid (C23:0; O), 3-oxodecanosanoic acid (C22:1; O), 2-hydroxypropyl stearate, and furan fatty acids F6 (19FU-FA)]. We believe that these compounds’ abundance in tachyzoites treated with APi might be an indication of mitochondrial antioxidant enzymes’ failure to reverse the oxidative stresses induced on the PUFAs in intercellular parasites. Additionally, these induced peroxidants may affect cellular membrane lipids’ architecture and thus cause membrane permeability to other unwanted compounds leading to parasite death. However, these assumptions need further investigation.

Noteworthy is that APi strongly affected the production of 1-palmitoyl-lysophosphatidic acid, 1-eicosapentaenoyl-2-docosahexaenoyl-sn-glycero-3-phosphocholine, and cholesterol sulfate in intracellular parasites in a time-dependent manner. 1-Palmitoyl-lysophosphatidic acid is one of the crucial glycerophospholipids which is known to be involved in intracellular calcium ion mobilization and activation of phospholipase C (Watson et al., 1985). Therefore, we believe that the decrease in its production by APi treatment might be affecting the phospholipid biosynthesis and intracellular calcium homeostasis in the *T. gondii* parasite. Calcium is an important second messenger for *T. gondii* parasite lytic cycle operation (Arrizabalaga and Boothroyd, 2004; Triana et al., 2018). Thus, future studies will be needed to elucidate the effect of APi on calcium signaling and the committed enzymes involved in phospholipid and cholesterol synthesis that is crucial for the parasite survival and virulence in host cells (Arroyo-Olarte et al., 2015).

AZ is known to alter lipid synthesis, thus observing the decrease in 1-eicosapentaenoyl-2-docosahexanoyl-sn-glycero-3-phosphocholine was expected. However, in the case of APi, it was very interesting and will require future studies to understand APi’s effect on phospholipid production in *T. gondii* for possible identification of this compound and its derivatives as a phospholipid inhibitor.

Using Atov, we observed the production of 11-aminoundecanoic acid, 12-aminododecanoic acid, 13S-hydroxyoctadecadienoic acid, 14(S)-HDHA (14(S)-hydroxy docosahexaenoic acid), 16-feruloyloxypalmitic acid, 2-hydroxypropyl stearate, 3,6-anhydro-1-O-palmitoylhexitol, 3-(3,4-dihydroxyphenyl)propanoic acid, 3-hydroxybutyric acid, 3-hydroxynonanoic acid, 3-hydroxypentadecanoic acid, 3-hydroxytridecanoic acid, 2,3-dihydroxypropyl stearate, ethyl docosahexaenoate, ethyl oleate, ethyl palmitoleate, cis-12-octadecenoic acid methyl ester, 2-(acetylamino)hexanoic acid, 2-(2-amino-3-methylbutanamido)-3-phenylpropanoic acid, methyl 3-hydroxypalmitate, 1-hexadecyllysophosphatidylcholine, and 1-phenyl-1,3-octadecanedione in treated parasites. This suggests that the high ROS generated by Atov had induced oxidation of the high PUFA (hexaenoic acid, propanoic acid, octadecanoic, palmitic, stearic, pentadecanoic acid, nonanoic acid, undecanoic acid, arachidonic acid) found in the parasites. These oxidized lipids were different from those reported in APi. However, there were seven lipid-oxidized metabolites that were common to both APi- and Atov-treated groups. This confirmed our biochemical findings of both compounds generating high ROS and disrupting the mitochondria membranes of *T. gondii.*

It has been well documented that in *T. gondii*, there is only a single mitochondrion that provides its numerous-energetics requirement (Melo et al., 2000). Thus, our findings on APi and Atov disrupting the MMP points to the fact that the mitochondria of the parasite were affected, thereby affecting its antioxidant enzymatic machinery abilities to reverse the damage caused by APi and Atov treatment. This finding partially supports evidence that high ROS/MitoSOX production in tachyzoites causes distortion of the major cellular activities such as nucleic acid synthesis, protein synthesis, and lipid synthesis that support *T. gondii* gliding motility, attachment, invasion, survival, proliferation, and egress (Charvat and Arrizabalaga, 2016). Future molecular studies will be needed to completely understand this assumption.

## Conclusion

Taken together, our findings support other studies that reported that ROS inducers cause mitochondrial impairment in parasites, e.g., *Leishmania* spp (Mehta and Shaha, 2004; Das et al., 2008; Fonseca-Silva et al., 2011). Furthermore, excessive ROS production can directly or indirectly cause changes in calcium and other cations’ homeostasis and eventually parasite death (Mehta and Shaha, 2004; Das et al., 2008; Fonseca-Silva et al., 2011). Further studies will be required to understand whether APi has any implication on calcium signaling in *T. gondii* tachyzoites. Another limitation of our study is that we evaluated only ROS/MitoSOX’s effects, which is a small part of the several ROS encountered in cellular oxidative stress. Thus, we suggest further studies in other forms of ROS such as hydroxyl radical, carbonate radical anion, peroxynitrite, hypohalous acids, singlet oxygen, nitrogen dioxide radical, and hydrogen peroxide using both APi and Atov. Lastly, we discovered that APi and Atov caused elevated oxidative stress metabolite production as a result of high ROS/MitoSOX production exerted on tachyzoites *in vitro*. Our studies confirmed that our compound (APi) might have a similar mechanism of action to that of the primary drug (Atov), which is known to target mitochondrial membrane potential and also causes high production of ROS/MitoSOX in cancer cells and protozoan parasites.

## Data Availability

The original contributions presented in the study are included in the article/**Supplementary Material**. Further inquiries can be directed to the corresponding author.
