# Supplementary material for: Apigeninidin chloride disrupts Toxoplasma gondii Mitochondrial membrane potential and induce reactive oxygen species and metabolites production
Source: Front Cell Infect Microbiol. 2024 Nov 11;14:1368019. doi: 10.3389/fcimb.2024.1368019 (PMC11586383; doi:10.3389/fcimb.2024.1368019)
Supplement: Supplementary file 1 [file DataSheet1.docx]

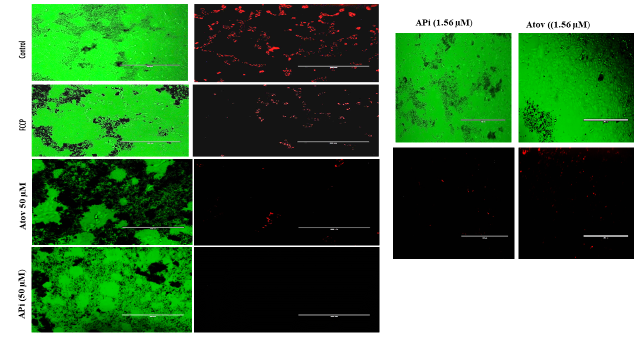


Supplementary Figure 1a showing the effect of Atovaquone (50 µM), FCCP (50 µM), APi (50 µM), and control (media only), on the mitochondria membrane potential in intracellular *Toxoplasma gondii* tachyzoites. and Figure 1b (1.56 µM), showed the effect of Atov and APi at low concentration intracellular. The green is representing compromised mitochondria and the red represents intact mitochondria of *T. gondii* tachyzoites. Short white arrows represent intracellular parasites. Images were captured with 20X using 400 µm
